# mTORC1 mediates fiber type-specific regulation of protein synthesis and muscle size during denervation

**DOI:** 10.1038/s41420-021-00460-w

**Published:** 2021-04-12

**Authors:** Jae-Sung You, Kookjoo Kim, Nathaniel D. Steinert, Jie Chen, Troy A. Hornberger

**Affiliations:** 1 Department of Comparative Biosciences in the School of Veterinary Medicine, University of Wisconsin-Madison, Madison, WI USA; 2 Department of Cell and Developmental Biology, University of Illinois at Urbana-Champaign, Urbana, IL USA

**Keywords:** Neurophysiology, TOR signalling

## Abstract

Skeletal muscle denervation occurs in diverse conditions and causes severe muscle atrophy. Signaling by mammalian target of rapamycin complex 1 (mTORC1) plays a central role in the maintenance of skeletal muscle mass by regulating net protein balance; yet, its role in denervation-induced atrophy is unclear. In this study, by using skeletal muscle-specific and inducible raptor knockout mice, we demonstrate that signaling through mTORC1 is activated during denervation and plays an essential role in mitigating the atrophy of non-type IIB muscle fibers. Measurements of protein synthesis rates of individual fibers suggest that denervation increases protein synthesis specifically in non-type IIB muscle fibers and that mTORC1 is required for this event. Furthermore, denervation induced a more pronounced increase in the level of phosphorylated ribosomal S6 protein in non-type IIB muscle fibers than in type IIB muscle fibers. Collectively, our results unveil a novel role for mTORC1 in mediating a fiber type-specific regulation of muscle size and protein synthesis during denervation.

## Introduction

Skeletal muscle is a highly plastic tissue that comprises different types of muscle fibers whose mass and size largely depend on neural activity. Loss of neural activity or denervation occurs in a variety of conditions, including nerve injury, diseases, and aging, and leads to severe muscle atrophy through the negative balance between protein synthesis and degradation^1,2^. Denervation-induced muscle atrophy contributes to weakness, loss of independence, and an increased risk of morbidity and mortality; however, its molecular regulators remain poorly defined.

The mammalian target of rapamycin complex 1 (mTORC1) is one of the key regulators of cell size and is also implicated in denervation-induced atrophy. Quy et al.^3^ first reported that signaling through mTORC1 is not only activated by denervation, but is also required for increasing protein synthesis and counteracting the development of atrophy. Since then, Tang et al.^4^ concluded that denervation-induced mTORC1 signaling actually promotes atrophy through negative feedback inhibition of Akt and a subsequent increase in the expression of atrogenes such as MAFbx E3 ubiquitin ligase. While these studies revealed contrasting roles of mTORC1 by using the same mTORC1 inhibitor rapamycin, MacDonald et al.^5^ did not find any effect of rapamycin on denervation-induced muscle atrophy. Hence, the role of mTORC1 in denervation atrophy remains controversial.

The inconsistency of the aforementioned results could be due to different efficacy/actions of systemic rapamycin administration in vivo. For instance, Tang et al.^4^ showed the expected inhibition of mTORC1 and increase in Akt phosphorylation by rapamycin in denervated muscle whereas, in the study by MacDonald et al.^5^, rapamycin appeared to inhibit mTORC2 more than mTORC1, leading to decreased Akt phosphorylation during denervation. Thus, in the current study, we set out to circumvent the potential issues that are associated with the use of rapamycin by using mice with skeletal muscle-specific and inducible knockout of raptor, the defining component of mTORC1.

## Results

### Signaling through mTORC1 contributes to the regulation of ubiquitin-mediated proteolytic pathway during denervation

**Fig. 1:**
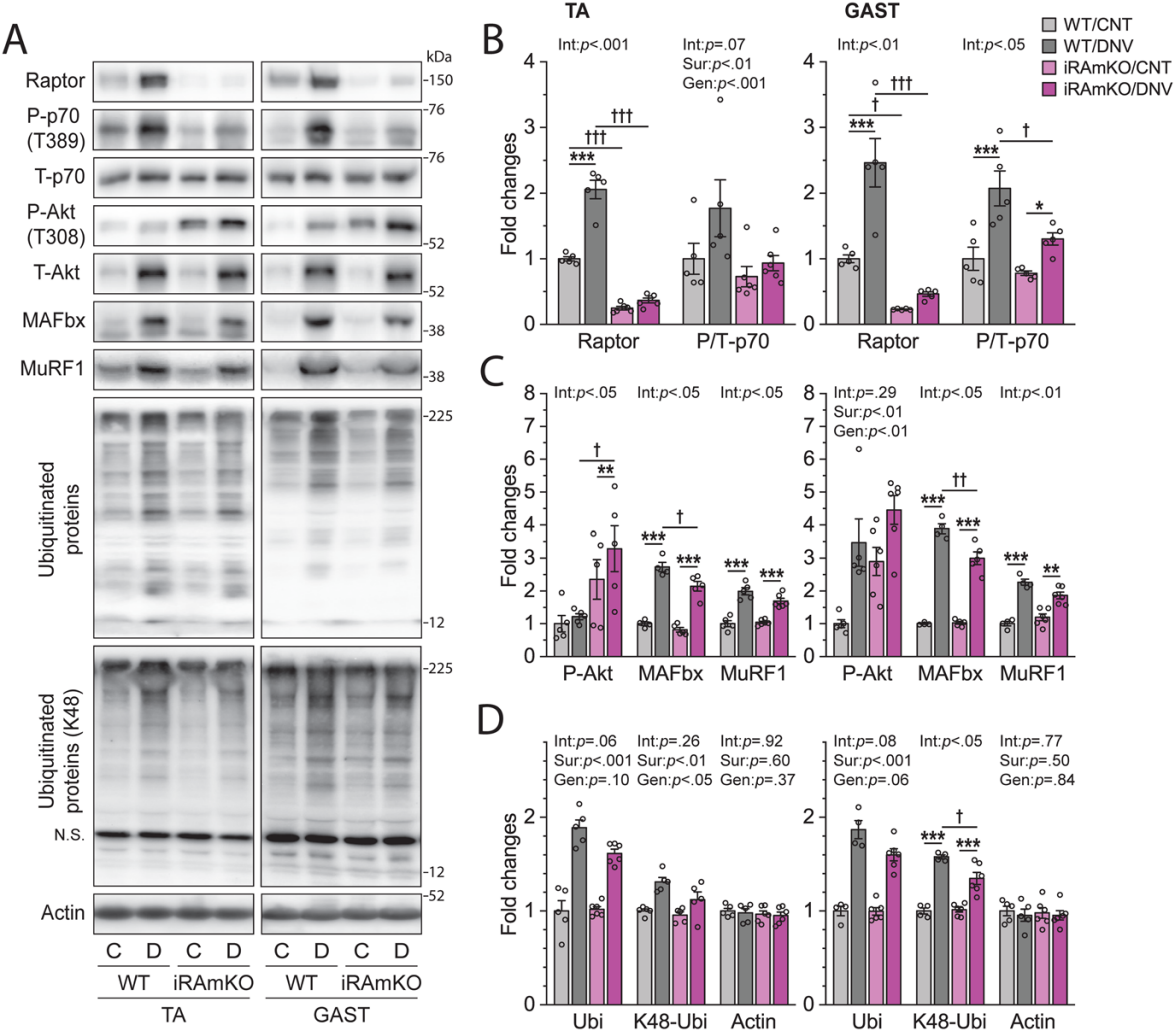
Signaling through mTORC1 contributes to the regulation of ubiquitin-mediated proteolytic pathway during denervation. **A** Skeletal muscle-specific inducible raptor knockout (iRAmKO) mice and control littermates (WT) were subjected to denervation (right leg, D or DNV) and sham (contralateral left leg, C or CTL) surgeries. Tibialis anterior (TA) and gastrocnemius (GAST) muscles were collected 7 days after surgery and analyzed by western blotting for the indicated proteins. N.S., non-specific band. **B**–**D** The levels of raptor and phosphorylated (P)/total (T) p70 ratio (**B**), P/T Akt ratio, MAFbx, and MuRF1 (**C**), and global ubiquitination (Ubi), K48-linked ubiquitination (K48-Ubi), and actin (**D**), were quantified and expressed relative to WT/CTL. *n* = 4–6 mice/group. Data are presented as mean ± s.e.m. Surgery effects (Sur, *p < 0.05, **p < 0.01, ***p < 0.001), genotype effects (Gen, ^†^*p* < 0.05, ^††^*p* < 0.01, ^†††^*p* < 0.001), and interaction between Sur and Gen (Int), by two-way mixed ANOVA.

We have previously shown that the loss of raptor protein in inducible raptor knockout (iRAmKO) mice was maximal within 14–21 days of being treated with tamoxifen^6^. Hence, to determine the role that raptor/mTORC1 exerts during denervation, we subjected iRAmKO and littermate wild-type (WT) mice to unilateral denervation at 21 days after tamoxifen treatment. At 7 days post denervation, both raptor protein and mTORC1 signaling as revealed by the phosphorylation of the 70 kDa ribosomal S6 protein (p70) on the threonine 389 residue were increased in tibialis anterior (TA) and gastrocnemius (GAST) muscles from WT mice when compared to the contralateral muscles from the same mice (Fig. 1A, B). As expected, the increased levels of raptor and mTORC1 signaling were reduced in the muscles of the iRAmKO mice (Fig. 1A, B). Consistent with the ability of mTORC1 to inhibit signaling through Akt, the reduced mTORC1 signaling in iRAmKO mice was associated with an enhanced amount of phosphorylated Akt (i.e., active Akt) and suppressed expression of MAFbx in denervated muscles (Fig. 1A, C and Fig. S1). Likewise, iRAmKO modestly but significantly attenuated K48 linkage-specific poly-ubuiquitination (i.e., ubiquitin-mediated proteolysis) in denervated muscles (Fig. 1A, D). Combined, these results indicate that signaling through mTORC1 contributes to the regulation of the ubiquitin-mediated proteolytic pathway during denervation.

### Signaling through mTORC1 alleviates atrophy of non-type IIB muscle fibers, but not of type IIB fibers, during denervation

**Fig. 2:**
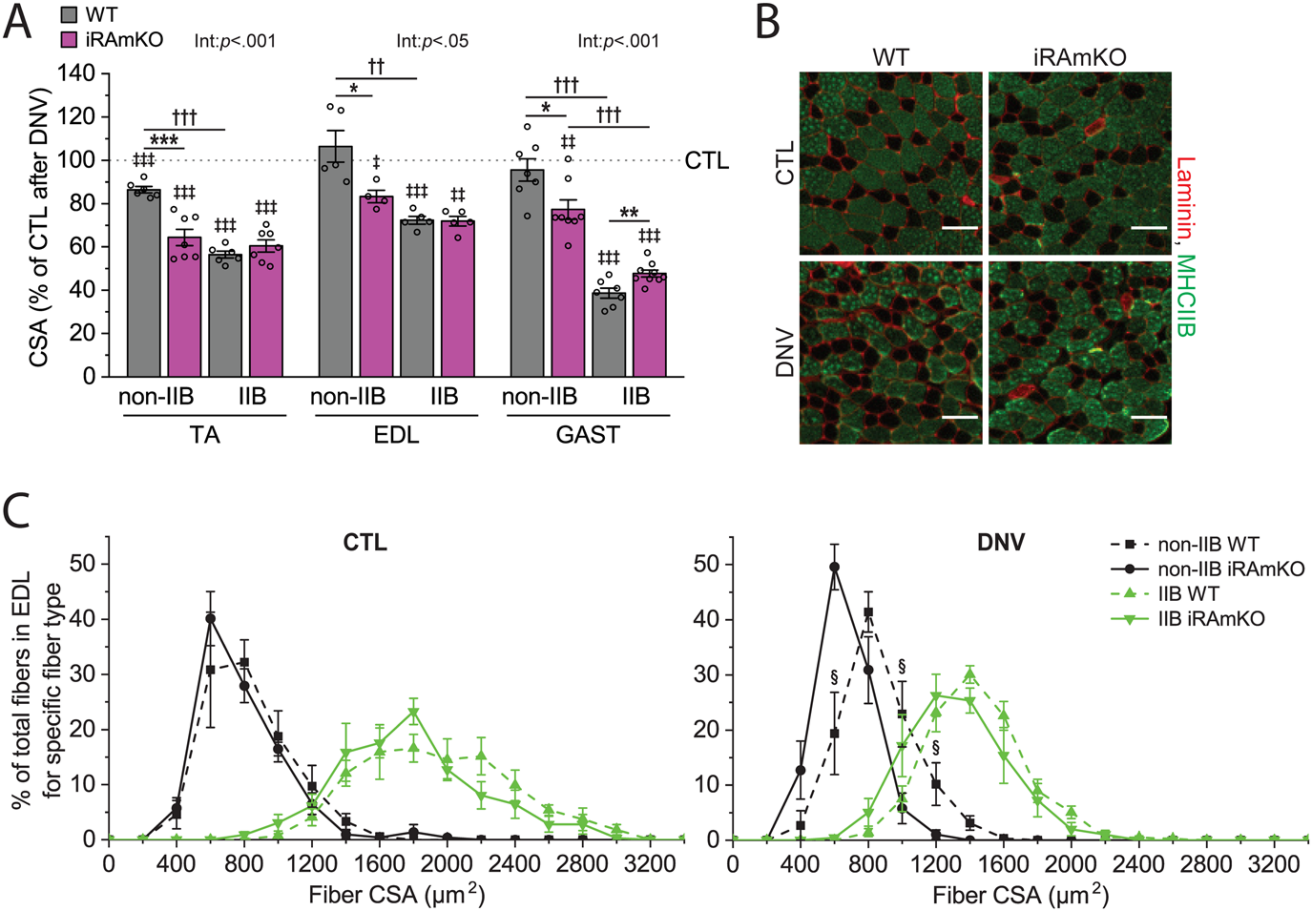
Signaling through mTORC1 alleviates atrophy of non-type IIB muscle fibers, but not of type IIB fibers, during denervation. **A** Skeletal muscle-specific inducible raptor knockout (iRAmKO) mice and control littermates (WT) were subjected to denervation (right leg, DNV) and sham (contralateral left leg, CTL) surgeries. Tibialis anterior (TA), extensor digitorum longus (EDL), and gastrocnemius (GAST) muscles were collected 14 days after surgery and analyzed by immunohistochemistry for cross-sectional area (CSA) of type IIB and non-type IIB muscle fibers. Type IIB muscle fibers were identified by type IIB myosin heavy chain (MHCIIB). **B**, **C** Representative cross-sectional images (**B**) and fiber CSA distribution (**C**) of the denervated and CTL muscles (EDL) from WT and iRAmKO mice. Scale bar: 70 µm. *n* = 4–9 mice/group. Data are presented as mean ± s.e.m. Surgery effects (^‡^*p* < 0.05, ^‡‡^*p* < 0.01, ^‡‡‡^*p* < 0.001) by paired *t* test. Genotype effects (*p < 0.05, **p < 0.01, ***p < 0.001), fiber type effects (^††^*p* < 0.01, ^†††^*p* < 0.001), and interaction between genotype and fiber type effects (Int), by two-way mixed ANOVA. ^§^*P* ≤ 0.05 vs. iRAmKO by unpaired *t* test.

The activation of mTORC1 signaling that occurs during denervation could either alleviate or exacerbate muscle atrophy depending on its impact on the balance between protein synthesis and degradation^3,4^. The anabolic/catabolic response of muscle fibers to atrophic conditions can be fiber type-specific^7,8^. Hence, it is possible that the anabolic/catabolic balance and resulting atrophic response, which is impacted by mTORC1 signaling during denervation, could also be fiber type-specific. Accordingly, we analyzed the size of type IIB (non-human muscle fiber type) and non-type IIB (human muscle fiber types) muscle fibers from three different muscles collected 14 days after denervation. Consistent with a recent report^9^, type IIB fibers of WT TA muscles underwent more severe atrophy than their non-type IIB fibers (Fig. 2A). In extensor digitorum longus (EDL) and GAST muscles, the non-type IIB fibers did not even show a significant reduction in size (Fig. 2A, B). Interestingly, however, the atrophy resistance of non-type IIB muscle fibers was largely lost in muscles from the iRAmKO mice, while type IIB fibers were either unaffected or even slightly protected from atrophy by the loss of raptor (Fig. 2A–C). Together, these results identify a fiber type-specific role of mTORC1 signaling that, depending on the fiber type, can either attenuate or augment denervation-induced atrophy.

### Signaling through mTORC1 enhances protein synthesis rates and the level of phosphorylated S6, preferably in non-type IIB muscle fibers

**Fig. 3:**
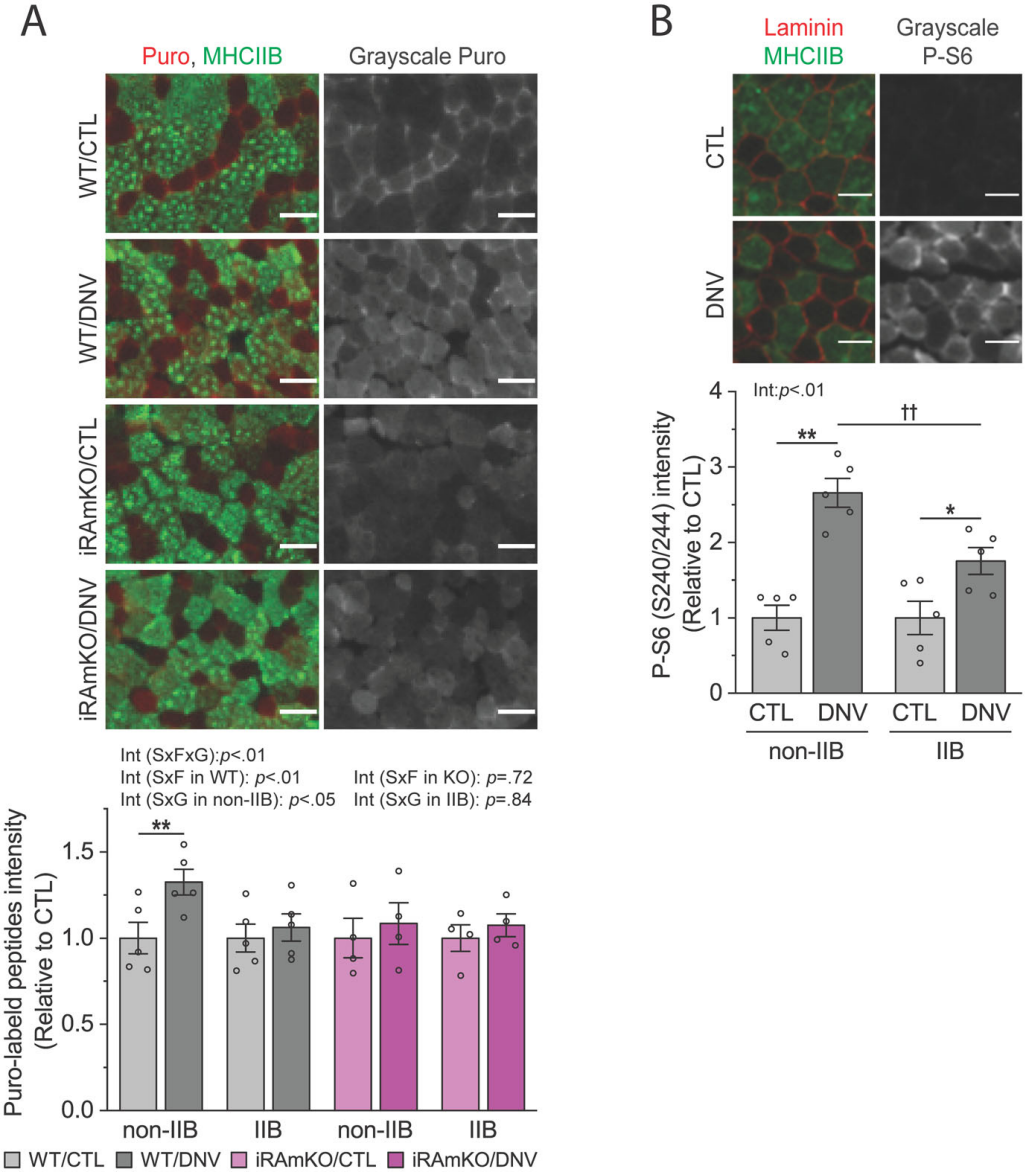
Signaling through mTORC1 enhances protein synthesis and the level of phosphorylated S6 preferably in non-type IIB muscle fibers. **A**, **B** Skeletal muscle-specific inducible raptor knockout (iRAmKO) mice and control littermates (WT) were subjected to denervation (right leg, DNV) and sham (contralateral left leg, CTL) surgeries. At 14 days post surgery, the mice were injected with puromycin, and extensor digitorum longus (EDL) muscles were collected 30 min after the injection. The samples were analyzed by immunohistochemistry for the total intensity of puromycin-labeled peptides (Puro) per type IIB and non-type IIB muscle fiber cross-section (CS) (**A**) or for the total intensity of phosphorylated (P) S6 protein per type IIB and non-type IIB muscle fiber CS (**B**). Type IIB muscle fibers were identified by type IIB myosin heavy chain (MHCIIB). Note that EDL muscles from the right and left legs were frozen together in OCT for immunohistochemistry on the same slide. Scale bar: 50 µm (**A**) or 30 µm (**B**). *n* = 4–5 mice/group. Data are presented as mean ± s.e.m. Surgery effects (*p < 0.05, **p < 0.01), fiber type effect (^††^*p* < 0.01), and interaction (Int) among surgery (S), fiber type (F), and genotype (G) effects, by three- and two-way mixed ANOVA.

Because iRAmKO promoted atrophy of non-type IIB muscle fibers during denervation despite the overall suppression of the ubiquitin proteolytic pathway, we asked if iRAmKO inhibited protein synthesis in denervated non-type IIB muscle fibers. As such, we examined the rate of protein synthesis at the single fiber level using an immunohistochemical version of the surface sensing of translation (SUnSET)^10^. Interestingly, the results revealed that the rate of protein synthesis was increased only in non-type IIB muscle fibers following denervation and that this increase was abolished by the loss of raptor (Fig. 3A). It has been reported that the phosphorylation of ribosomal protein S6, a downstream target of mTORC1, can play a critical role in the maintenance of cell size independently of protein synthesis^11,12^. To determine the potential involvement of S6 phosphorylation in the fiber type-specific regulation of muscle size during denervation, we also examined the amount of phosphorylated S6 in each fiber type. The results showed that denervation induced an increase in the level of phosphorylated S6 in non-type IIB muscle fibers and that this increase was significantly attenuated in type IIB muscle fibers (Fig. 3B). Combined, these findings suggest that, during denervation, mTORC1 enhances protein synthesis and the level of phosphorylated S6, preferably in non-type IIB muscle fibers, which possibly confers the atrophy-resistant property of these fibers.

### Signaling through mTORC1 regulates muscle fiber type proportion during denervation

**Fig. 4:**
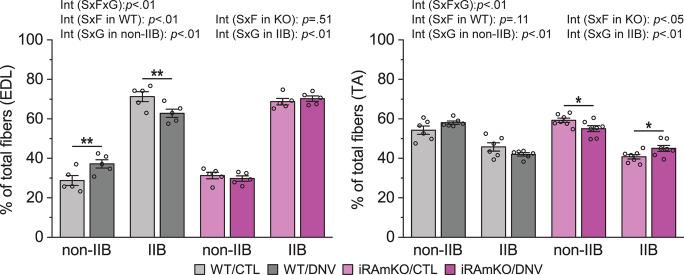
Signaling through mTORC1 regulates muscle fiber type proportion during denervation. Skeletal muscle-specific inducible raptor knockout (iRAmKO) mice and control littermates (WT) were subjected to denervation (right leg, DNV) and sham (contralateral left leg, CTL) surgeries. Extensor digitorum longus (EDL) and tibialis anterior (TA) muscles were collected 14 days after surgery and analyzed by immunohistochemistry for the percentage of type IIB and non-type IIB muscle fibers. Type IIB muscle fibers were identified by type IIB myosin heavy chain (MHCIIB). *n* = 5–7 mice/group. Data are presented as mean ± s.e.m. Surgery effects (*p < 0.05, **p < 0.01) and interaction (Int) among surgery (S), fiber type (F), and genotype (G) effects, by three- and two-way mixed ANOVA.

In addition to protein metabolism, mTORC1 appears to regulate muscle fiber type proportion during denervation as forced activation mTORC1 by muscle-specific deletion of its inhibitor TSC1 (TSCmKO) was shown to decrease the proportion of non-type IIB muscle fibers following denervation^9^. Based on this finding, we expected that the inactivation of mTORC1 by iRAmKO would lead to an increased proportion of non-type IIB muscle fibers following denervation. However, the loss of raptor counteracted the denervation-induced increase in the proportion of non-type IIB muscle fibers in EDL, and even decreased the non-type IIB muscle fiber proportion in the TA following denervation (Fig. 4). Although unexpected, this mTORC1-dependent increase/maintenance of more oxidative non-type IIB muscle fibers during denervation is in line with the role of mTORC1 positively regulating skeletal muscle oxidative function^13^.

## Discussion

Whether the enhanced signaling through mTORC1 that occurs during denervation impacts the development of atrophy has been unclear. There have been attempts to directly address this question by using constitutive skeletal muscle raptor knockout (RAmKO) mice^9,14^. In those studies, RAmKO enhanced the development of atrophy, suggesting that denervation-induced mTORC1 signaling may prevent gross muscle atrophy. However, those results were potentially confounded by the dystrophic nature of the RAmKO muscle^15^. On the other hand, acute deletion of skeletal muscle raptor in iRAmKO mice did not induce any of the problematic traits shown in the RAmKO mice^6,16^. In this study, by using iRAmKO mice, we demonstrate that signaling through mTORC1 alleviates only the atrophy of non-type IIB muscle fibers. We also found that mTORC1 signaling promotes protein synthesis specifically in non-type IIB muscle fibers during denervation, providing mechanistic insights into the fiber type-specific regulation of muscle fiber size by mTORC1.

Another interesting finding in this study is that, contrary to non-type IIB muscle fibers, type IIB fibers of GAST muscle were partially protected from denervation atrophy by iRAmKO, and this effect was associated with the protection of the denervation-induced loss of GAST muscle mass (Fig. S2) [note that GAST muscle is predominantly comprised of type IIB muscle fibers (~74%)^17^, making its mass highly sensitive to changes in type IIB muscle fiber size]. Although we did not focus on the potential mechanisms of the anti-atrophic effect of iRAmKO on type IIB muscle fibers, a recent study by Lang et al.^7^ provided an important clue. Specifically, they performed single muscle fiber proteomics and found that denervation upregulates many proteasomal subunits predominantly in type IIB muscle fibers. This finding first suggests that, in addition to the lack of increase in protein synthesis, a high rate of proteasome-mediated proteolysis in type IIB muscle fibers resulted in a more severe atrophic response to denervation than other fiber types. Furthermore, because mTORC1 is also known to promote proteolysis through increased gene expression of proteasomal subunits^18^, inhibition of mTORC1 by iRAmKO may have suppressed the type IIB muscle fiber-specific increase in proteasomal degradation and thereby the severe atrophy of that fiber type from GAST during denervation.

Although our results indicate that denervation-induced increase in mTORC1 signaling protected non-type IIB muscle fibers from atrophy, it is questionable whether this role of mTORC1 is indeed functionally beneficial. This suspicion mostly derives from the inhibitory function of mTORC1 on autophagy^19^, which is critical for the maintenance of muscle integrity^20^. Indeed, studies have shown that denervation suppresses autophagy flux, and forced activation of mTORC1 by TSCmKO leads to a severe myopathy during denervation^3,9^. However, contrary to the results of our study, deletion of autophagy gene, *Atg7*, or TSCmKO aggravated denervation-induced muscle atrophy^9,20^, particularly in non-type IIB fibers^9^, which appears to be a phenotypic consequence of severe autophagy inhibition. Furthermore, it has been shown that prolonged denervation (>7 days) overcomes mTORC1-mediated autophagy suppression and even increases autophagy flux by upregulating autophagy inducers^11^. Although still unclear, these results suggest that the spontaneous (unforced) activation of mTORC1 signaling we observed at 14 days after denervation would not have suppressed autophagy flux to the level that deteriorates the integrity of non-type IIB muscle fibers.

The current study generates several follow-up questions: how denervation differentially regulates mTORC1-mediated protein synthesis among fiber types, whether denervation also regulates proteolysis in a fiber type-specific manner via mTORC1, and whether the fiber type-specific role of mTORC1 alters in different types of denervation such as that it occurs during aging. Addressing these issues will be of great importance for future therapies targeting skeletal muscle atrophy.

## Materials and methods

### Animals

iRAmKO male mice were generated by crossing female mice homozygous for floxed raptor (The Jackson Laboratory, Bar Harbor, ME, USA) with male mice homozygous for floxed raptor and hemizygous for human skeletal actin-driven expression of a mutated estrogen receptor-flanked Cre recombinase (HSA-MCM)^6^. The presence of the HSA-MCM allele was determined by PCR genotyping with tail snips^21^, and littermates that were homozygous for floxed raptor but did not contain the HSA-MCM allele served as WT controls. To induce the translocation of MCM into the nucleus, both iRAmKO and WT mice at 6 weeks of age were injected intraperitoneally with a tamoxifen solution every 24 h for 5 days as previously described^6^. All mice were housed in a room maintained with a 12-h light/dark cycle and received food and water ad libitum. Mice aged 2.5 months old were randomly allocated to experimental groups and received surgical procedures under anesthesia with isoflurane. All of the methods used in this study were approved by the Institutional Animal Care and Use Committee at the University of Wisconsin-Madison.

### Denervation

Unilateral denervation of the hindlimb muscles was performed as previously described^22^. Briefly, a 0.5-cm section of the sciatic nerve was removed from the right leg, while the left leg was subjected to a sham surgery and served as a contralateral control. After the surgery, the skin incision was closed with a 5-0 polysorb suture.

### Measurements of protein synthesis

In vivo measurements of protein synthesis were performed with the SUnSET technique^10^. Specifically, puromycin dissolved in phosphate-buffered saline (PBS) was injected intraperitoneally into mice at the concentration of 0.04 µmol/g body weight, and muscles were collected exactly 30 min after injection. The amount of puromycin-incorporated peptides was then analyzed by immunohistochemistry as below.

### Western blotting

Frozen muscles were homogenized with a Polytron in ice-cold buffer [40 mM Tris (pH 7.5), 1 mM EDTA, 5 mM EGTA, 0.5% Triton X-100, 25 mM β-glycerolphosphate, 25 mM NaF, 1 mM Na_3_VO_4_, 10 mg/ml leupeptin, and 1 mM PMSF], and protein concentrations of the whole homogenates were determined with the DC Protein Assay Kit (Bio-Rad, Hercules, CA, USA). Western blotting was performed with an equal amount of protein from each whole homogenate sample following the standard procedure as previously described^23^. Images of the blots were captured with a Chemi410 camera mounted to an Autochemisystem (UVP, Upland, CA, USA) and quantified densitometrically by ImageJ 1.53 (NIH, Bethesda, MD, USA; https://imagej.nih.gov/ij/).

### Immunohistochemistry

Immunohistochemical analyses of cross-sectional area, protein synthesis, and phosphor-S6 were performed as previously described^22,24^. Briefly, muscles from denervated and sham-operated contralateral legs were submerged individually (for cross-sectional area) or adjacent to one another (for protein synthesis/phosphor-S6) in optimal cutting temperature compound at resting length and frozen in liquid nitrogen-chilled isopentane. Cross-sections (10-μm-thick) from the mid-belly of the muscles were fixed in acetone for 10 min, rehydrated with PBS for 15 min, and incubated for 1 h in PBS containing 0.5% bovine serum albumin, 0.5% Triton X-100, and anti-mouse IgG Fab (for protein synthesis; 1:10, 115-007-003, Jackson ImmunoResearch, West Grove, PA, USA) or 5% normal goat serum (for phosphor-S6; 005-000-121, Jackson ImmunoResearch). The target proteins on the sections were then probed with the appropriate antibodies and visualized with a DS-QiMc camera mounted on an 80i epifluorescence microscope (Nikon, Melville, NY, USA). All image analyses were performed by investigators who were blinded to the sample identification.

### Antibodies

The primary antibodies used in this study are anti-raptor (#2280), anti-phospho-p70 (T389, #9234), anti-p70 (#2708), anti-phosphor-Akt (T308, #9275), anti-Akt (#9272), anti-K48 linkage-specific polyubiquitin (#8081), anti-pan-actin (#8456), anti-phosphor-S6 (S240/244, #5364) from Cell Signaling Technology (Danvers, MA, USA); anti-ubiquitin (sc8017) from Santa Cruz Biotechnology (Dallas, TX, USA); anti-laminin (L9393), anti-puromycin (MABE343) from MilliporeSigma (Burlington, MA, USA); anti-MAFbx, anti-MuRF1 from Regeneron Pharmaceuticals Inc. (Tarrytown, NY, USA); anti-type IIB myosin heavy chain from the Developmental Studies Hybridoma Bank (Iowa City, IA, USA).

### Quantitative PCR (qPCR)

Frozen muscles were homogenized with a ribonuclease-free pestle in ice-cold TRIzol (Invitrogen, Carlsbad, CA, USA). Total RNA was extracted and reverse transcribed using the GeneJET RNA Purification Kit (Thermo Scientific, Waltham, MA, USA) and the qScript cDNA Synthesis Kit (Quanta Bioscience, Beverly, MA, USA), respectively. Complementary DNA was subjected to qPCR on a StepOnePlus Real-Time PCR System (Applied Biosystems, Foster City, CA, USA) using SYBR Green. The primer sequences used were as follows: 5′-CTTTCAACAGACTGGACTTCTCGA-3′ (forward) and 5′-CAGCTCCAACAGCCTTACTACGT-3′ (reverse) for *MAFbx*, 5′-GAGAACCTGGAGAAGCAGCT-3′ (forward) and 5′-CCGCGGTTGGTCCAGTAG-3′ (reverse) for *MuRF1*, and 5′-GCAGCCTCGTCCCGTAGAC-3′ (forward) and 5′-ATGGCAACAATCTCCACTTTGC-3′ (reverse) for *Gapdh* as an internal control.

### Statistics

All values were presented as mean ± s.e.m. with individual animal data points shown in graphs (the number of the points represents *n*). The sample size for each experiment was chosen based on previous publications and preliminary data. Mice exhibiting any sign of abnormality determined by pre-established criteria (e.g., lethargy, elevated respiration rate, reduced body weight, etc.) were excluded from experiments. Statistical significance (*p* < 0.05) was determined by a two-tailed paired or unpaired *t* test, or two-/three-way mixed analysis of variance, followed by a Tukey’s or planned pairwise comparisons. All statistical analyses were performed upon verification of the test assumptions using the OriginPro 2019 software (OriginLab, Northampton, MA, USA) and R 4.0.2 (R Core Team). The variation between the test groups was similar.

## Supplementary information

Supplementary Figure 1.

Supplementary Figure 2.
