# Supplementary figures and images for: mTORC1 mediates fiber type-specific regulation of protein synthesis and muscle size during denervation
Source: Cell Death Discov. 2021 Apr 12;7:74. doi: 10.1038/s41420-021-00460-w (PMC8042034; doi:10.1038/s41420-021-00460-w)

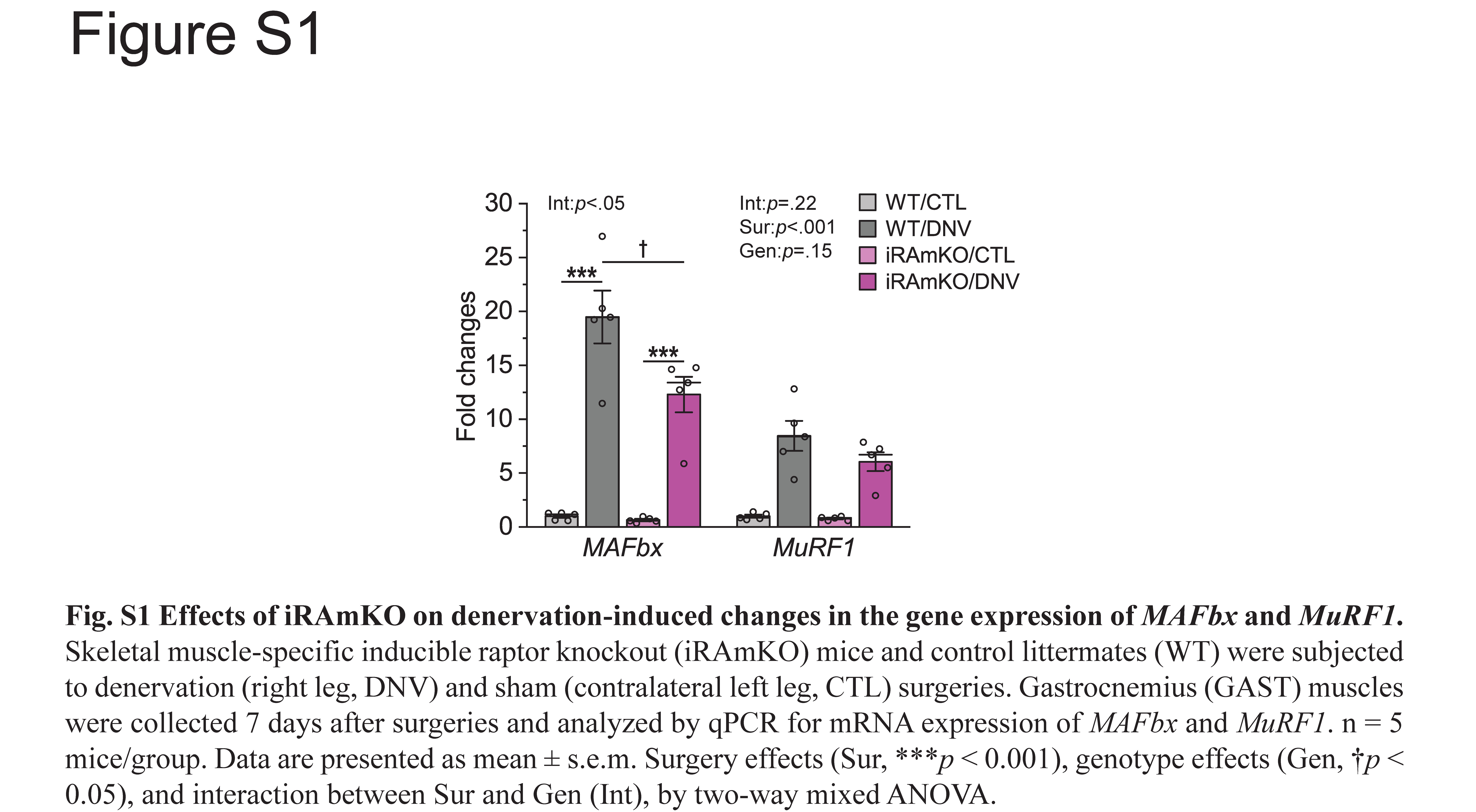

Supplement: Supplementary file 1 — Supplementary Figure 1. [file 41420_2021_460_MOESM1_ESM.png]

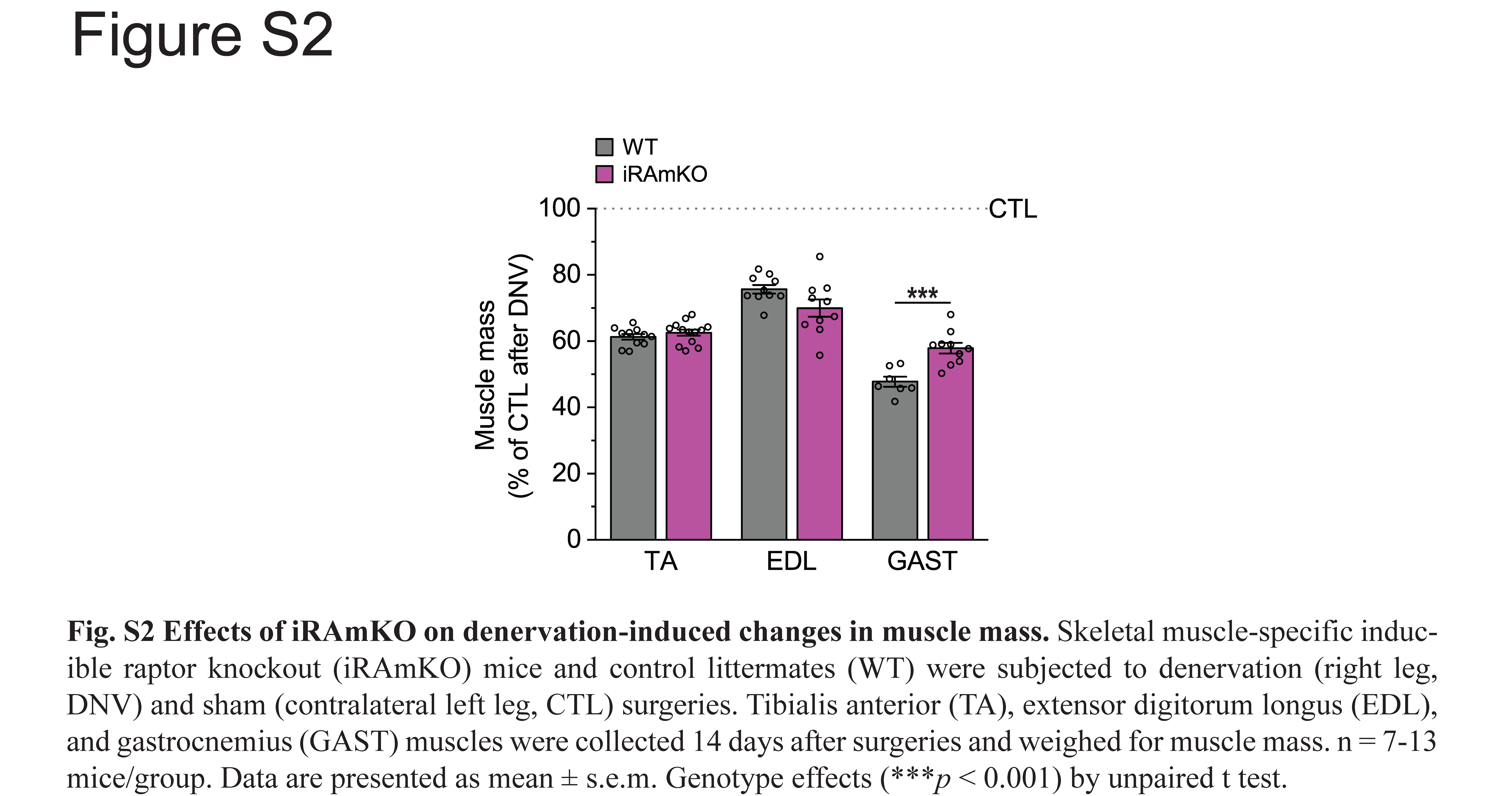

Supplement: Supplementary file 2 — Supplementary Figure 2. [file 41420_2021_460_MOESM2_ESM.png]
